# Effects of mycophenolate mofetil on key pattern of coronary restenosis: a cascade of in vitro and ex vivo models

**DOI:** 10.1186/1471-2261-5-9

**Published:** 2005-05-12

**Authors:** Rainer Voisard, Sandra Viola, Verena Kaspar, Christian M Weber, Lutz von Müller, Regine Baur, Iris Gastrock-Balitsch, Vinzenz Hombach

**Affiliations:** 1 Department of Internal Medicine II – Cardiology, Institute of Mikrobiology and Immunology, University of Ulm, Germany; 2 Department of Virology, Institute of Mikrobiology and Immunology, University of Ulm, Germany

## Abstract

**Background:**

Mycophenolate mofetil (MMF), the prodrug of mycophenolic acid (MPA), is a rationally designed immunosuppressive drug. The current study investigates the effect of MMF on key pattern of restenosis in a cascade of in vitro and ex vivo models.

**Methods:**

Part I of the study investigated in northern blot and cytoflow studies the effect of MMF (50, 100, 150, 200, 250, and 300 μg/mL) on TNF-α induced expression of intercellular adhesion molecule 1 (ICAM-1) in human coronary endothelial cells (HCAEC) and human coronary medial smooth muscle cells (HCMSMC). Part II of the study applied a human coronary 3D model of leukocyte attack, the 3DLA-model. HCAEC and HCMSMC were cultured on both sides of a polycarbonate filters, mimicking the internal elastic membrane. Leukocyte attack (LA) was carried out by adding human monocytes (MC) on the endothelial side. The effect of MMF (50 μg/mL) on adhesion and chemotaxis (0.5, 1, 2, 3, 4, 6, and 24 h after LA) and the effect on proliferation of co-cultured HCMSMC (24 h after LA) was studied. In part III of the study a porcine coronary organ culture model of restenosis (POC-model) was used. After ex vivo ballooning MMF (50 μg/mL) was added to the cultures for a period of 1, 2, 3, 4, 5, 6, and 7 days. The effect on reactive cell proliferation and neointimal thickening was studied at day 7 and day 28 after ballooning.

**Results:**

Expression of ICAM-1 in northern blot and cytoflow studies was neither clearly inhibited nor stimulated after administration of MMF in the clinical relevant concentration of 50 μg/mL. In the 3DLA-model 50 μg/mL of MMF caused a significant antiproliferative effect (p < 0.001) in co-cultured HCMSMC but had no effect on MC-adhesion and MC-chemotaxis. In the ex vivo POC-model neighter reactive cell proliferation at day 7 nor neointimal hyperplasia at day 28 were significantly inhibited by MMF (50 μg/mL).

**Conclusion:**

Thus, the data demonstrate a significant antiproliferative effect of clinical relevant levels of MMF (50 μg/mL) in the 3DLA-model. The antiproliferative effect was a direct antiproliferative effect that was not triggered via reduced expression of ICAM-1 or via an inhibition of MC-adhesion and chemotaxis. Probably due to technical limitations (as e.g. the missing of perfusion) the antiproliferative effect of MMF (50 μg/mL) could not be reproduced in the coronary organ culture model. A cascade of focused in vitro and ex vivo models may help to gather informations on drug effects before large experimental studies are initiated.

## Background

Stent coating with immunosuppressive or cytostatic agents are valid advances in the struggle against restenosis following coronary intervention. However these therapies are hampered by high costs, especially in the case of multivessel disease. Moreover it is not entirely clear whether restenosis is merely delayed and not inhibited [[1], review]. Consequently the intense search for a systemic approach to inhibit restenosis is required.

Restenosis is essentially characterized by migration and proliferation of smooth muscle cells and extracellular matrix accumulation. However, there is now increasing evidence for a role of inflammation in the development of restenosis. The underlying molecular mechanisms of restenosis are, in fact, most probably regulated by inflammatory mediators, such as cytokines [[2], review]. The situation resembles to a certain degree to the activation of the immune system during organ rejection. Therefore it is not surprising that immunosuppressive agents are potential candidates in the treatment of restenosis.

Mycophenolate mofetil (MMF), the prodrug of mycophenolic acid (MPA), is a rationally designed immunosuppressive drug. The active metabolite MPA is a selective, non-competitive and reversible inhibitor of inosine monophosphate dehydrogenase (IMPDH) and of the type II isoform in particular [3]. The primary mechanism of action of MPA is presumed to be anti-lymphoproliferative, a result of inhibition of inosine 5'-monophosphate dehydrogenase (IMPDH), which is required for the de novo synthesis of guanosine nucleotides which are necessary for DNA and RNA synthesis and for lymphocytes to proliferate maximally after stimulation [4]. Antiproliferative effects of MMF have been described in non-immune cells [[5], review].

In the past data of animal studies could not be transferred easily to the clinical situation due to species differences. In the current study a cascade of human in vitro models in combination with a porcine coronary ex vivo model is applied to investigate the effects of MMF on key pattern of restenosis. Unexpected or conflicting data can be analysed before large experimental studies are initiated. Furthermore attention is focused on the relation between significant inhibitory effects in vitro (SI) and maximal plasma levels in vivo (MPL), the SI/MPL-ratio [6].

Central part of the current study is a 3D human coronary transfilter co-culture model of leukocyte attack [7]. In this model the effect of MMF on monocyte (MC) adhesion and chemotaxis and reactive cell proliferation of co-cultured smooth muscle cells (SMC) are investigated. In order to obtain information on the effect of MMF on TNF-alpha induced expression of adhesion molecules, the effect on expression of ICAM-1 is studied in northern blot and cytoflow studies. Finally MMF is added in a porcine coronary organ culture model of restenosis for 1, 2, 3, 4, 5, 6, and 7 days and the effect on reactive cell proliferation and neointimal hyperplasia is investigated.

## Methods

### Cell culture

Human coronary endothelial cells (HCAEC) and human coronary smooth muscle cells (HCMSMC) were purchased at Cambrex Bio Science (Vervier, B). HCAEC were cultured in Endothelium Growth Medium (Cambrex) and identified by the typical "cobble stone" growth pattern and positive reaction against von Willebrand factor (Dakopatts). HCMSMC were grown in Smooth Muscle Cell Growth Medium (Cambrex). For identification of HCMSMC antibodies against smooth muscle α-actin (Renner, Darmstadt, D) were used. Human MC were isolated from the residual leukocytes of single donors using MACS cell-isolation kit (Milteny Biotec GmbH).

### Mycophenolate Mofetil

Mycophenolate mofetil (MMF): Cellcept^®^, Roche, Basel, CH, 0.005 – 500 μg/mL, dilution: aqua ad inject., MPL: 34 μg/mL [8].

### RNA extraction and Northern blot analysis

For Northern blot studies of the effect of MMF/TNF-α treatment on expression of ICAM-1, monocultures of HCAECs and HCMSMCs were incubated with MMF (50, 100, 150, 200, 250, and 300 μg/mL) for a period of 18 hrs. During the last 6 hrs of MMF incubation, expression of adhesion molecules was stimulated by adding of TNF-α (20 ng/mL). Total RNA (3 × 10^6 ^cells) was isolated with RNEasy Mini Kit (Qiagen), and 10 μg of RNA was used in standard Northern blot analysis with an ICAM-1 probe.

A non radioactive labelling and detection system (Amersham Biosciences Europe GmbH, Freiburg, D) was used to detect the relative band density of ICAM-1 mRNA in comparison with TNF-α-stimulated cells. GAPDH was used as a control. Experiments were performed in triplicate.

### Flow cytometry

For flow cytometry analysis of the expression of ICAM-1 in HCAEC and HCMSMC cells were trypsinized and seeded into 6-well dishes (5 × 10^4 ^cells). MMF (50, 100, 150, 200, 250, and 300 μg/mL) was added to the cultures for a period of 18 hrs. During the last 6 hrs of MMF incubation, the expression of adhesion molecules was stimulated by adding of TNF-α (20 ng/mL).

After MMF/TNF-α treatment, cells were washed twice with phosphate-buffered saline (pH 7.2) and trypsinized. Cells were resuspended in 100 μL of a FITC-conjugated monoclonal antibody directed against ICAM-1 (clone 84H10, Dianova Immunotech; final concentration 10 μg/mL) and incubated for 20 min at 4°C. A total of 1 × 10^4 ^cells (100% gated) were analyzed immediately with a flowcytometer (BDFACsCalibur, Durchflußzytometer Becton Dickinson, Heidelberg, D).

The effects of MMF (50 μg/mL – 300 μg/mL) on vitality of HCAEC and HCMSMC were analyzed with propidium iodide (Sigma-Aldrich, Taufkirchen, D).

### The 3DLA model

Three-dimensional human coronary units of leukocyte attack (3DLA units) mimic the inner layers of human coronary arteries [7]. The internal elastic membrane is represented by a polycarbonate filter with a thickness of 10 μm and a pore size of 5 μm (Whatman, Göttingen, D). Filters were fixed in a specially designed frame and inserted in a siliconized culture dish. On both sides of the filters cell cultures were established, direct contact of the cultures was made possible through the pores of the filter.

HCMSMC were seeded on one side of the filter at a density of 2.5 × 10^4 ^cells / cm^2^. After 24 h cells had attached to the surface and frame and filters were turned upside down. HCAEC were seeded on the opposite side of the filter at a density of 2.5 × 10^4 ^cells / cm^2^. Both HCAEC- and HCMSMC-cultures were supplied with the appropriate culture medium and cultured for 14 days.

At day 14, 3DLA-units were incubated with MMF (50 μg/mL) for 18 h. During the last 6 h of MMF incubation, the models were treated with TNF-α (20 ng/mL). For leukocyte attack, the required numbers of MC was calculated in relation to the relative concentration of MC in the full human blood [7]. 3 × 10^5 ^MC were seeded on the endothelial side of the 3DLA-units. The effect of MMF on MC-adhesion and MC-chemotaxis was studied at 30 min, 1, 2, 3, 4, 6, and 24 h after leukocyte attack; the effect on proliferation of co-cultured HCMSMC was investigated 24 h after leukocyte attack. Controls were performed without MMF-treatment. [for detailed information: [7]].

### The porcine coronary organ culture model

Fresh hearts of 12 pigs, ranging in age from 3 to 5 months, weighing 100 to 120 kg, were obtained from a local slaughterhouse. In the laboratory, the left anterior descending coronary artery (LAD) was carefully prepared. Section were made at 4 mm intervals perpendicular to the vessel wall axis [9].

#### Ex vivo ballooning and adding of MMF

For ex vivo angioplasty the prepared LAD segments were placed over a 3 mm balloon catheter (Medtronic 14K2030E, Medtronic, Kerkrade, North Carolina, USA) and were treated with 9 bar for a period of 60 s. After ex vivo ballooning MMF (50 μg/mL) was added to the cultures for a period of 1, 2, 3, 4, 5, 6, and 7 days. At each medium exchange the drug was renewed.

#### Cultivation and fixation of coronary organ cultures

After ballooning the segments were transferred to six-well plates (Tecnomara, Fernwald, D) and cultured in a mixture of Waymouth's MB 752/1 and Ham F12 nutrient mixture (1:1; vol/vol; Cambrex) supplemented with 15% fetal calf serum (Cambrex) at 37°C in 5% carbon dioxide. Organ cultures were cultured for 7 and 28 days, culture medium was exchanged every second or third day. Culture conditions for control groups were exactly the same as described for the angioplasty/MMF group.

#### Analysis of reactive cell proliferation and neointimal thickening

The effect of MMF (50 μg/mL) on reactive cell proliferation and neointimal thickening was studied at day 7 and day 28 after ballooning, controls were performed with and without ballooning [for detailed information: [9]].

### Statistical analysis

Data of northern blot and flow cytometry studies were presented as mean ± S.D. Statistical significance of differences between controls and drug-treated cells was determined by paired Student's t-test. The Mann-Whitney rank-sum test was used to investigate the significance of differences in the 3DLA-model and the organ culture model. Statistical significance was accepted for *P *< 0.05.

## Results

### Identification of cells

In monocultures of HCAECs cells were identified by a positive reaction with antibodies directed against von Willebrand factor and by the typical "cobblestone" growth pattern in culture. Monocultures of HCMSMC exhibited the "hill and valley" growth pattern and reacted positively with antibodies against smooth muscle α-actin.

### Effect of MMF on ICAM-1 mRNA levels: Northern blot studies

**Figure 1:**
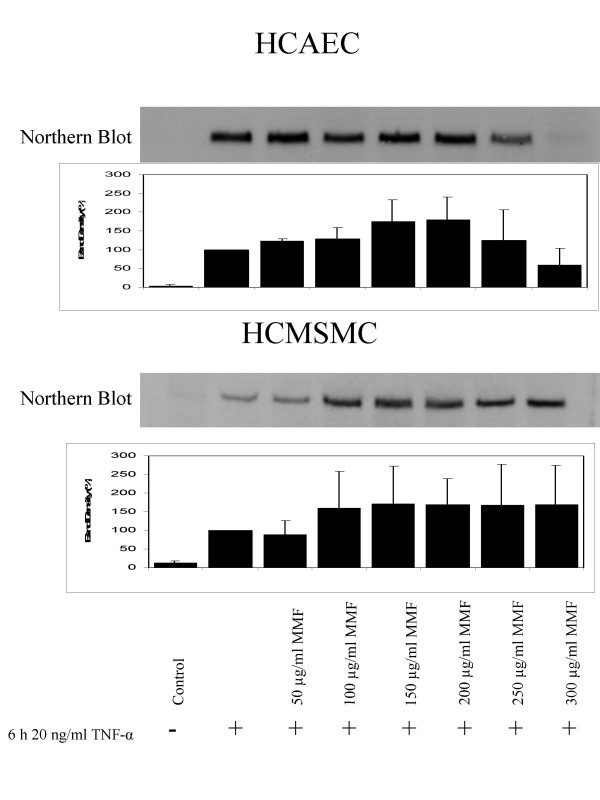
Northern blots and relative band densities of TNF-α-induced expression of ICAM-1 mRNA after incubation of HCAECs and HCMSMCs with 50, 100, 150, 200, 250, and 300 μg/mL of MMF.

After TNF-α stimulus, band density of mRNA ICAM-1 in HCAEC was increased 30-fold, which corresponds to a relative band density of 100% (Fig. 1). After incubation of HCAEC with MMF in concentrations of 50, 100, 150, 200, and 250 μg/ml expression of ICAM-1 was further increased by 23.92% (p = 0.01), 29.51% (n.s.), 75.87% (n.s.), 79% (n.s.), and 24.34% (n.s.). MMF in a concentration of 300 μg/mL caused an inhibition of ICAM-1 expression by 40.57% (n.s.).

In HCMSMC, band density of mRNA ICAM-1 was increased 7.5-fold after TNF-α-stimulus, relative band density was increased from 13.4% to 100% (n.s.). Incubation with MMF in the concentration of 50 μg/mL caused a slight inhibitory effect on band density of mRNA ICAM-1 by 12.02% (n.s.). After incubation of HCMSMC with MMF in concentrations of 100, 150, 200, 250, and 300 μg/mL relative band density of ICAM-1 was increased by 59.9% (n.s.), 70.61% (n.s.), 68.7% (n.s.), 67.8% (n.s.), and 69.4% (n.s.).

Both in HCAEC and HCMSMC, expression of GAPDH after adding of MMF in concentrations of 50, 100, 150, 200, and 250 μg/ml was identical with untreated controls.

### Effect of MMF on ICAM-1: Flow cytometry studies

**Figure 2:**
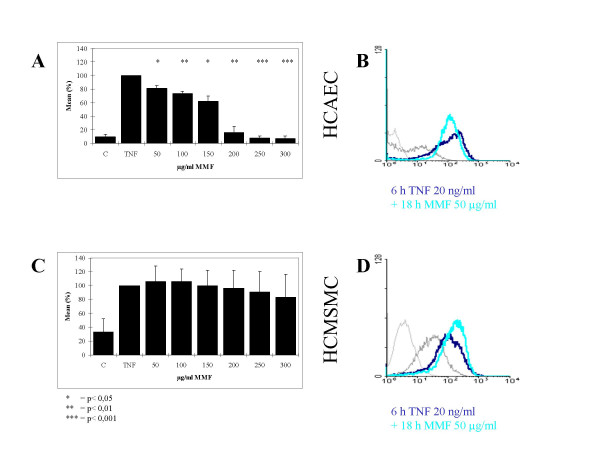
Graphics and histograms (cytoflow data) of the effect of MMF (50, 100, 150, 200, 250, and 300 μg/mL) on TNF-α-induced expression of ICAM-1 in HCAECs (A, B) and HCMSMCs (C, D) after 18 h. Negative control (dotted line) and ICAM-1 expression in untreated cells (grey line) are included.

The effects of MMF (50, 100, 150, 200, 250, and 300 μg/mL) on the TNF-α induced expression of ICAM-1 are demonstrated in Figure 2. A dose dependent significant inhibition of ICAM-1 expression was detected in HCAEC. No significant effect was seen in HCMSMC.

In HCAEC, treatment with TNF-α increased the mean fluorescence levels (%) of ICAM-1 expression 10.5-fold from 9.48% to 100.00%. Incubation of HCAEC with MMF caused a dose dependent inhibition of ICAM-1 expression. After incubation with MMF in concentrations of 50, 100, and 150 μg/mL expression of ICAM-1 was significantly decreased by 18.57%, 26.46%, and 37.92% (p < 0.05, p < 0.01, p < 0.05). Incubation with 200, 250, and 300 μg/mL caused an decrease by 83.77%, 92.41%, and 93.17% (p < 0.01, p < 0.001, p < 0.001).

In HCMSMC a very weak inhibition of ICAM-1 expression was detected without statistical significance. After incubation of HCMSMC with MMF in concentrations of 50, 100, 150, and 200 μg/mL no inhibitory effect on ICAM-1 expression was detected. MMF in concentrations of 250 and 300 μg/mL caused a 9.03% (n.s.) and 16.68% (n.s.) inhibition of ICAM-1 expression.

In HCAEC no toxic effects were detected after adding of MMF in concentrations of 50 μg/ml, 100 μg/ml, and 150 μg/ml, little toxic effects were found after adding of MMF in concentrations of 200 μg/ml, 250 μg/ml, and 300 μg/ml. In HCMSMC no toxic effects were detected after adding of MMF in concentration of 50 μg/ml – 300 μg/ml.

### 3DLA-Model: Effect of MMF on monocyte adhesion, chemotaxis, and proliferation of human coronary smooth muscle cells

**Figure 3:**
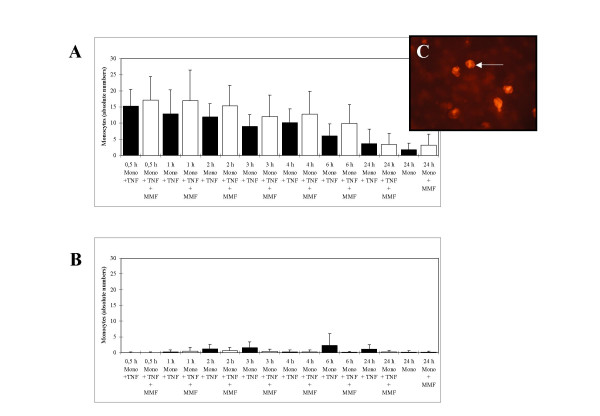
(A) Effect of MMF (50 μg/mL) on monocyte adhesion after leukocyte attack on the endothelial side of the human coronary transfilter co-culture model (3DLA units). (B) Effect of MMF (50 μg/mL) on monocyte chemotaxis after leukocyte attack on the endothelial side of the human coronary transfilter co-culture model (3DLA units). (C) Identification of monocytes by positive staining against CD68 (arrow).

In 3DLA units the effect of MMF in a concentration of 50 μg/mL on monocyte adhesion, chemotaxis, and proliferation of HCMSMC was studied. 3DLA-units were successfully established (Fig. 3). On the endothelial side of the units one to two layers of cells were found. The superficial layer of these cells was composed of HCAEC, as identified by positive reaction with antibodies directed against von Willebrand factor. On the HCMSMC side of the units, three to five layers of cells with the typical hill and valley growth pattern were observed.

Human monocytes were isolated from the residual leukocytes of single donors and identified by positive reaction with antibodies directed against CD68. A 93% purity of monocyte preparations was determined by flow cytometry.

Adhesion of MC was slightly stimulated 0.5, 1, 2, 3, 4 and 6 h after leukocyte attack in MMF-treated 3DLA-units (Fig. 3A and 3C). In comparison to 3DLA-units without MMF treatment the stimulatory effect was 12.6% (n.s.), 31.5% (p < 0.05), 29.2% (p < 0.05), 33.2% (n.s.), 24.9% (n.s.), and 64,1% (p = 0.002). 24 h after leukocyte attack a very small inhibitory effect of MMF by 8.1% (n.s.) was detected.

Chemotaxis of MC from the endothelial side of the model to the HCMSMC side of the model was very little, both with and without MMF-treatment (Fig. 3B).

**Figure 4:**
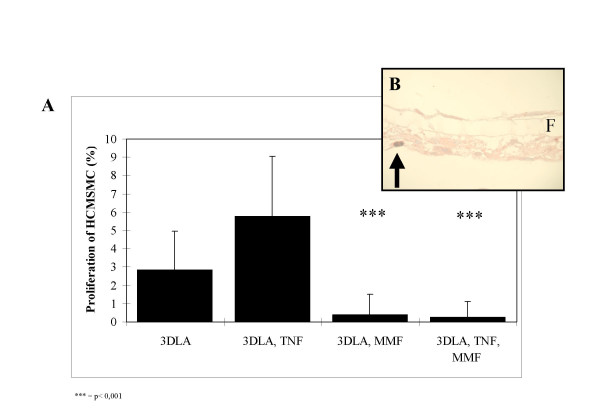
(A) Effect of MMF (50 μg/mL) on proliferative activity of co-cultured HCMSMC after leukocyte attack with monocytes on the endothelial side of TNF-α-stimulated 3DLA units. (B) Proliferation of smooth muscle cell demonstrated by positive staining of against BrdU (arrow). F = Filter, *** = p < 0.001.

Proliferation of HCMSMC in the transfilter co-culture units was significantly inhibited by MMF (50 μg/ml) by more than 90% (Fig. 4A and 4B). Proliferation of HCMSMC in the 3DLA-units after TNF-α stimulus was significantly stimulated by 103% (p < 0.001) in comparison to untreated controls. Adding of MMF in a concentration of 50 μg/mL significantly inhibited proliferation of HCMSMC by 95.5% (p < 0.001). The inhibitory effect was not influenced by TNF-α. In the transfilter co-culture units without TNF-α the inhibitory effect in comparison to control was 93.4% (p < 0.001).

### Coronary Organ Culture-Model: Effect of MMF on cell proliferation and neointimal proliferation after ex vivo ballooning

**Figure 5:**
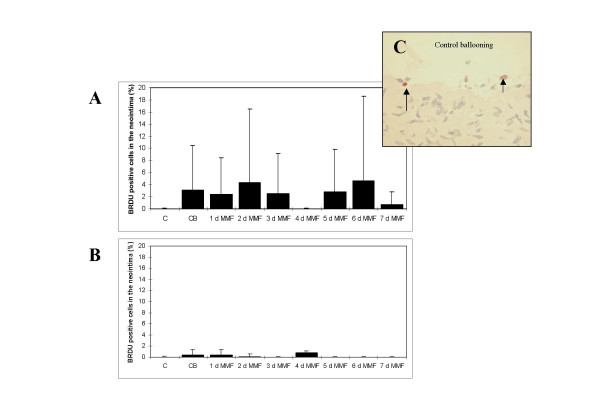
Effect of a 1–7 days treatment with MMF (50 μg/mL) on reactive cell proliferation in balloon-injured porcine coronary organ cultures at day 7 (A) and day 28 (B). C = control, CB = control ballooning. Positive staining against BrdU (arrow) in the ballooning control group (C).

**Figure 6:**
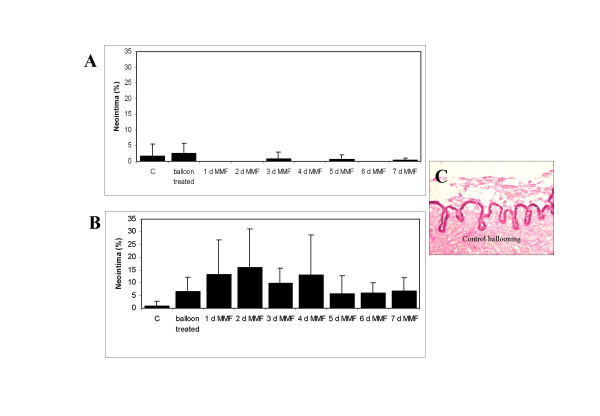
Effect of a 1–7 days treatment with MMF (50 μg/mL) on reactive neointimal hyperplasia in balloon-injured porcine coronary organ cultures at day 7 and day day 28. C = control, CB = control ballooning. Elastica van Gieson-staining in the ballooning control group (C).

The effects of ex vivo ballooning in the porcine organ culture model have been recently characterized by our group (9). Maximal reactive cell proliferation was detected at day 7, maximal reactive neointimal hyperplasia was found at day 28. In the current study the effect of a 1, 2, 3, 4, 5, 6, and 7 days incubation with MMF (50 μg/mL) on reactive cell proliferation (Fig. 5) and neointimal hyperplasia was studied 7 (Fig. 6A) and 28 days (Fig. 6B) after ex vivo ballooning. No clear inhibitory or stimulatory effect was detected.

At day 7 neointimal cell proliferation was slightly increased (n.s.) in comparison to untreated controls. Adding of MMF for 1, 3, and 5 days did not exhibit an effect on cell proliferation. A stimulatory effect was found after adding of MMF for a period of 2 and 6 days, an inhibitory effect was seen after adding of MMF for a period of 4 and 7 days. 28 days after ballooning cell proliferation in the POC-model was very low, both in untreated controls and after treatment with MMF. Almost no cell proliferation was detected in the media.

At day 7 after ex vivo ballooning neointimal hyperplasia was very low in comparison to controls. No neointimal hyperplasia was detected after adding of MMF for 1, 2, 4, and 6 days, an inhibitory effect on neointimal hyperplasia was seen after adding of MMF for a period of 3, 5, and 7 days. 28 days after ex vivo ballooning neointimal hyperplasia was increased by 809% (n.s.). Adding of MMF for a period of 1, 2, 3, 4, and 7 days caused an increase of neointimal thickening by 108.9%, 144%, 51.8%, 104.9%, and 4.5%. Adding of MMF for a period of 5 and 6 days decreased neointimal hyperplasia by 9.2% and 7.7%. Due to high standard deviations statistical significance was not achieved.

## Discussion

The present study employes a cascade of in vitro and ex vivo models to investigate the effect of MMF on key processes of coronary restenosis. Three basic conclusions of the study were determined. First, therapeutical concentrations of MMF exhibit a significant antiproliferative effect in HCMSMC. Second, this antiproliferative effect was not triggered via inhibition of adhesion and chemotaxis of MC or reduced expression of ICAM-1 on mRNA or protein levels. Third, a significant antiproliferative effect of MMF could not be reproduced in the porcine coronary ex vivo model.

MPA, a product of Penicillium fungus, was originally isolated in 1896, and shown to have anti-neoplastic, anti-viral, anti-fungal and immunosuppressive activity. MMF is the semi-synthetic morpholinoethyl ester of MPA. After oral administration and absorbtion of MMF, the ester linkage is rapidly hydrolyzed by esterases to yield MPA, the active immunosuppressive agent. The bioavailability of oral MPA from MMF is 96% and the maximum of plasma concentration occurs about 2 h after administration [[10], review]. Over 10 years ago, MMF was first shown to prolong organ allograft survival [11]. Additional early studies demonstrated that MMF prevents acute rejection, reverses acute rejection and increases graft survival in several species and in different animal transplantation studies. Shortly thereafter, the first human renal allograft recipients were treated with MMF [[12], review]. Although many data exist on MMF in organ transplantation, data of the effect of MMF in experimental models of atherosclerosis and restenosis are very limited [13,14].

The group of Fraser-Smith [13] demonstrated that orally administered MMF inhibits restenosis after carotid injury in a rat model and Raisanen-Sokolowski et al. [14] reported that MMF inhibits inflammation and SMC proliferation in a rat aortic allograft model. In these studies the relation between the significant in vitro respectively experimental effect (SI) and the maximal plasma level (MPL), the SI/MPL-ratio [6] was smaller than one, indicating an at least theoretical clinical relevance of the data. Recently the antiproliferative properties of MMF in non-immune cells have been summarized in a valuable review [5]. Gregory et al. [15] reported that human aortic smooth muscle cell proliferation was significantly reduced by MMF in the presence of angiotensin II or β-FGF and Mohacsi et al. [16] demonstrated that MMF potently inhibited rat and human aortic smooth muscle cell proliferation.

In accordance with these reports the current study demonstrates a significant inhibition of HCMSMC-proliferation after incubation with 50 μg/mL of MMF in the 3DLA-model. The SI/MPL-ratio [6] of 1.47 indicates that this concentration is merely slightly above systemic plasma levels of MMF. The complexity of the 3DLA-model allows studies of HCMSMC-proliferation and MC-adhesion and MC-chemotaxis in one model. Although an inhibitory effect of MMF on CD4/CD8-lymphocyte proliferation is described in the literature and an inhibitory effect on MC-adhesion and chemotaxis might have been expected with reference to the clinical successes of the agent in the therapy of acute organ rejection [11], no inhibitory effect on MC-adhesion and MC-chemotaxis was detected in the 3DLA-model. An inhibitory effect on MC-adhesion and chemotaxis would have been of importance due to the fact that during the first 24 h after leukocyte attack monocytes seem to play a predominant role in comparison to CD4/CD8-lymphocyte attack, as recently demonstrated by our group [7]. The data indicate that the strong antiproliferative effect of MMF was not triggered via an inhibitory effect on MC.adhesion or chemotaxis.

It has been reported that MMF inhibited the induced expression of adhesion molecules on endothelial cells measured by marked antibodies and scanning fluorimetry [17]. On the other hand the group of Raab et al. [18] demonstrated that MMF in a concentration 10 μM (corresponding to 3.2 μg/mL) does not inhibit TNF-α-induced stimulation of ICAM-1 in HUVEC. In the current study conflicting data are reported on mRNA and protein levels. Although in northern blot studies a stimulatory effect of MMF on TNF-α-induced expression of ICAM-1 was found, a dose dependent inhibitory effect was detected in flow cytometry studies. Due to the fact that littly toxic effects were found after incubation of HCAEC with MMF in concentrations of 200 μg/ml – 300 μg/ml, the decrease of ICAM-1 protein levels in HCAEC may be partially explained by these toxic effects. Clinically relevant concentrations of MMF (50 μg/mL) however neighter stimulated nor inhibited expression of ICAM-1. If a concentration of 50 μg/mL of MMF is considered the data are in accordance with the reports of Raab et al. [18]. Due to the fact that MMF (50 μg/mL) did not inhibit expression of ICAM-1 on mRNA- and protein levels and exhibited no effect on MC-adhesion and MC-chemotaxis, the described antiproliferative effect of MMF eighter a direct one or it was triggered via other pathways not investigated in the current study.

With the hypothesis of a direct antiproliferative effect MMF (50 μg/mL) was studied in the coronary porcine organ culture system of restenosis, the POC-model [9]. In the POC-model we have previously described a maximal reactive cell proliferation at day 7 and a maximal reactive neointimal hyperplasia at day 28. In order to get information on the period of time needed to treat MMF was added for a period of 1, 2, 3, 4, 5, 6, and 7 days, the time span between angioplasty and the peak of reactive cell proliferation. In the POC-model reactive cell proliferation was inhibited after adding of MMF for a period of 4 days and 7 days, no inhibitory effect on neointimal hyperplasia was detected. These results are in accordance with the data of the experimental models applied by Fraser-Smith et al. [13] and Raisanen-Sokolowski et al. [14], describing an antiproliferative effect of an about 5-times increased concentration of MMF. Surprisingly in the current study no antiproliferative effect was detected after adding MMF for a period of 5 days and 6 days. Due to the fact that the ester linkage of MMF is rapidly hydrolysed in the plasma to MPA [10], it can be excluded that an inactive form of MMF caused the missing inhibitory effect. However high standard deviations and the absence of perfusion in the model may have contributed to the negative effect. In the presented coronary ex vivo model of restenosis [9,19] the solved drug gets into contact with the adventitial side, the endothelial side, and both frontal sides of the artery segment. Therefore our group has reported earlier that the model mimics a simultaneous intra/extravascular drug administration [19]. However due to the absence of perfusion the contact between the endothelial side of the artery segments and the culture medium is limited. Limited nutrition of this area may be critical because ballooning injury and reactive cell proliferation&neointimal hyperplasia are expected to occure predominantly in this region of the vessel wall.

## Conclusion

The current data demonstrate a significant antiproliferative effect of MMF in concentrations close to the systemic plasma level (SI/MPL-ratio: 1.47). The effect was not triggered via inhibitory effects on expression of ICAM-1 or via inhibitory effects on MC-adhesion and MC-chemotaxis. Eighter the effect was a direct antiproliferative effect or it was triggered via pathways not investigated in the present study. Probably due to technical limitations (as e.g. the missing of perfusion) the antiproliferative effect of MMF (50 μg/mL) could not be reproduced in the coronary organ culture model. A cascade of focused in vitro and ex vivo models may help to gather informations on drug effects before large experimental studies are initiated.

## Competing interests

The author(s) declare that they have no competing interests.

## Authors' contributions

RV, RB, and VH designed the study, RV wrote the manuscript. VK carried out the cytoflow studies, northern blot studies were done by RB and IGB. CMW carried out the studies with the 3DLA-model, coronary organ culture studies were done by SV.

## Pre-publication history

The pre-publication history for this paper can be accessed here:


